# Superior functional outcome with dual mobility THR as compared to conventional THR in fracture neck femur: a prospective cohort study

**DOI:** 10.1051/sicotj/2021041

**Published:** 2021-08-17

**Authors:** Sanjay Agarwala, Ameya Katariya, Mayank Vijayvargiya, Vivek Shetty, Pravin Manohar Swami

**Affiliations:** 1 Chief of Surgery and Director Professional Services, P.D. Hinduja Hospital and Medical Research Centre Mumbai 400016 India; 2 Resident doctor, Department of Orthopedics, P.D. Hinduja Hospital and Medical Research Centre Mumbai 400016 India; 3 Junior Consultant, Department of Orthopedics, P.D. Hinduja Hospital and Medical Research Centre Mumbai 400016 India; 4 Consultant, Department of Orthopedics, P.D. Hinduja Hospital and Medical Research Centre Mumbai 400016 India; 5 Resident doctor, Department of Orthopedics, P.D. Hinduja Hospital and Medical Research Centre Mumbai 400016 India

**Keywords:** Fracture Neck Femur, Dual Mobility, Total Hip Replacement, Dislocation rates

## Abstract

*Introduction*: Total Hip Replacement (THR) in displaced Fracture Neck of Femur (FNOF) is associated with higher dislocation rates. Conventional THR with a large femoral head and anterior approach has reduced the instability, but it remains higher than THR done for other aetiology. Recent studies have shown reduced dislocation rates with dual mobility THR (DMTHR) for FNOF; however, there is a lack of comparative research to show its superiority over conventional THR. Further, its role in the Asian subcontinent, where the patient requires sitting cross-legged or squatting, has not been studied. *Methods*: A prospective cohort study of 103 elderly patients with displaced FNOF with a minimum follow-up of 1-year. Fifty-two patients were operated on with DMTHR and fifty-one patients with conventional THR. Both the groups were matched in terms of demographic data, surgical approach, and postoperative protocol. Radiological and functional outcomes in terms of Harris Hip Score (HHS), Range of motion, Patient Reported Outcome Measures (PROM), and Dislocation rate were compared between the two groups. *Results*: Mean HHS of the DMTHR group was 76.37 at three months and 87.02 at the end of the 1-year postoperatively, which was significantly better than the conventional THR group 65.65 at three months and 72.96 at 1-year. The range of motion was significantly better in the DMTHR group than the conventional THR group. There was no significant difference in radiological outcomes and postoperative dislocation rate between the two groups. *Conclusion*: Dual mobility implants give better results than conventional implants for primary THA in elderly patients of displaced FNOF regarding better function and greater range of motion.

## Introduction

THA is widely accepted as the treatment of choice for displaced fracture neck femur in the elderly population [1, 2]. Instability and postoperative dislocation remain among the major concerns and common causes of revision after conventional total hip arthroplasty [3]. The dislocation rate increases from 2–3% at early postoperative follow-up to 4.8% at 10 years and 7% at 25 years postoperatively [4, 5]. Factors influencing postoperative instability include age, activity level, history of previous surgery, soft tissue status, history of trauma, surgical approach, implant position, and diameter of the head and head-neck ratio [6–8].

Soft tissue and muscle around the hip play a vital role in the stability of the joint. A displaced fracture of the neck of the femur, being an acute traumatic event, results in its damage, making the patient prone to postoperative instability [9–11]. Increasing the head-neck ratio by increasing the head diameter (above 28 mm) and using an anterior or lateral approach reduces, to some extent, the potential for instability after THR [8, 12, 13].

The dual mobility design of THR implant, first introduced in 1974 by Gilles Bousquet, differs from conventional design in terms of having mobility at two different sites. This reduces the impingement, allows a larger head and greater range of motion [14, 15], and has lower postoperative dislocation rates. [16, 17] The enigma of early wear and intra prosthetic dislocation in the 1st generation implants was solved by 2nd and 3rd generations after incorporating optimal neck design (thin and with mirror polished surface), higher density poly, and larger retention collar [18–26]. Therefore, DMTHR has become an attractive option, especially in scenarios with a higher risk of instability like the displaced neck of femur fracture [3].

Literature [27–30] has shown the superiority of DMTHR for fracture neck of femur in reducing dislocation rates and requirement of Revision arthroplasty compared to conventional THR. However, studies that have assessed the difference in functional outcome between the two implant designs are lacking. In the Asian subcontinent, lifestyle and religious habits demand squatting, sitting on the floor, sitting cross-legged, and therefore requires higher degrees of range of motion. Thus, the difference in range of motion and ability to go back to pre-injury activity levels is of utmost importance, in between the DMTHR and conventional THR designs, in the Indian subcontinent.

Therefore, we hypothesize that displaced fracture neck of femur in elderly Indian population has better functional outcomes when primarily treated with DMTHR versus conventional THR. The present prospective cohort study aims at testing this hypothesis by comparing the short to mid-term clinical and radiological outcomes of these two groups.

## Materials and methods

**Figure 1:**
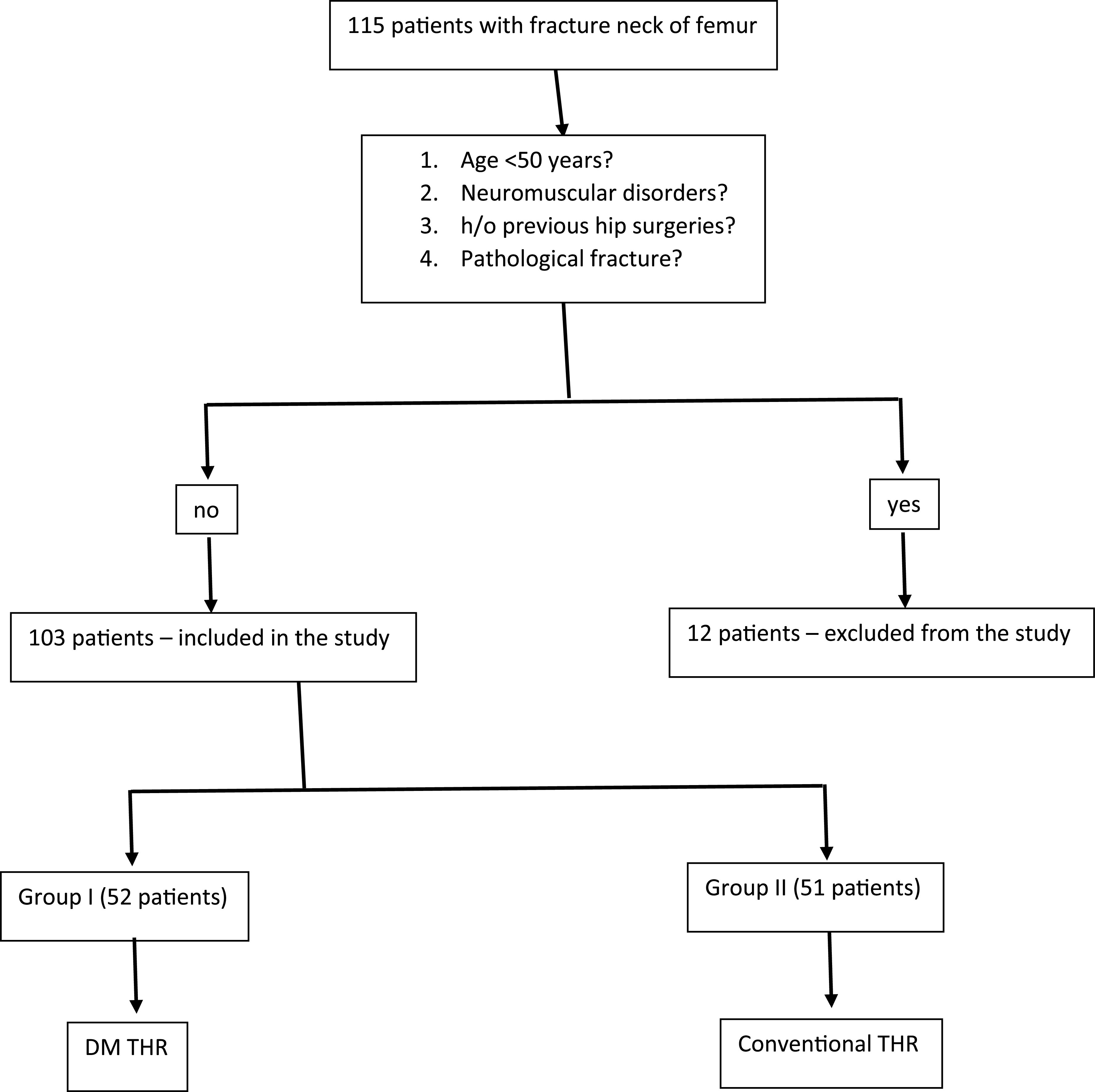
Flow diagram of the study.

The present study is a prospective cohort study conducted for one year from August 2018 to July 2019 at a tertiary care center. A total of 115 patients with displaced fracture neck of femur were included in the study. After excluding patients with age < 50 years, polytrauma, neuromuscular disorder, history of previous hip surgery, and the ones with pathological fractures, 103 patients participated in the study (Figure 1).

All the patients presented to the emergency department. After initial resuscitation, stabilization, and clinical assessment, patients were investigated with plain Anteroposterior and lateral radiographs to confirm the diagnosis. Patients were admitted and operated on for Total Hip Replacement by two different surgeons after a written and informed consent. Fifty-one patients, operated in the first six months, were offered conventional THR, and the remaining fifty-two, operated in the last six months, underwent DMTHR. The demographic data of patients in these two groups are detailed in Table 1.

**Table 1:** Patient demographics and distribution of implant used.

Variable	Conventional THR group (51 patients)	Dual mobility THR group (52 patients)	P-value
Gender			
Males	27(52.94%)	23(44.23%)	0.3788
Females	24(47.06%)	29(55.77%)	
Male/Female ratio	1.125	0.793	
Mean Age (years)	73.37	75.58	0.2124
Mean BMI (kg/m^2^)	25.13	24.92	0.7622
Profile of side among study cases			
Right	26 (50.98%)	28(53.85%)	0.7720
Left	25 (49.02%)	24(46.15%)	
ASA grade			
I	6 (11.77%)	6 (11.54%)	0.6939
II	25 (49.02)	30 (57.69%)	
III	17 (33.33%)	12 (23.08%)	
IV	3 (5.88)	4 (7.69)	
Mean surgical time (min)	58.3	60.2	0.6596
Surgical approach	Modified Hardinge	Modified Hardinge	
Average intra-operative blood loss (mL)	500	450	0.8077
Mean hospital stay (days)	3.83	4.02	0.4576
Number of patients that needed post op ICU	2	3	0.8836
Head size			
28 mm head	3 (5.88%)	52 (100%)	
32 mm head	20 (39.21%)	0	
36 mm head	28 (54.9%)	0	
Femoral stem:			
Cemented	24 cases (47.06%)	28 cases (53.85%)	0.6412
Uncemented	27 cases (52.94%)	24 cases (46.15%)	
Acetabulum			
Cemented	4 cases (7.84%)	6 cases (11.54%)	
Uncemented	47 cases (92.16%)	46 cases (88.46%)	0.2688

### Surgical technique

Preoperative templating was done in all the patients preoperatively, with the help of true size X-rays with size markers, to predict the implant size, position, and level of neck cut. All patients were operated in the lateral position, with a modified Hardinge approach, and in a similar operative environment. Evolutis Cupules CAPTIV® Dual Mobility Acetabular cups with spike and HACTIV® Sans collarette Avec calerett stems were used for all DMTHR. Whereas Zimmer Continuum® Acetabular System with CLS®/ CPT® 12/14 Femoral System or smith and nephew OXINIUM™ Femoral Heads 12/14 Taper with BASIS™ 12/14 Taper Primary Collared Stems Cobalt Chromium-ASTM F 75 was used for conventional THR. Details of implants and head sizes are mentioned in Table 1. Dual mobility cups were positioned according to the anatomy of the native acetabulum in such a way that there is no superior overhang to allow easy reduction [3]. Cemented or uncemented stem was used depending on bone quality and canal anatomy.

### Postoperative rehabilitation

All the patients had a similar rehabilitation protocol. Three doses, one preoperatively and two postoperatively of 1.5 g injectable cefuroxime, were given twelve hours apart. DVT prophylaxis was given in the form of mechanical (DVT stockings, pneumatic compression device) and pharmacological (aspirin or LMWH) methods. All patients were allowed full weight-bearing mobilization from day one and followed the same physiotherapy programme.

### Evaluation

All patients were followed up postoperatively at two weeks, six weeks, three months, twelve months, and yearly thereafter with a minimum follow-up of one year. HSS was calculated at three months and twelve months postoperatively. Patient-reported outcome measures (PROM), like the ability to do daily activities, were also assessed. Squatting and sitting cross-legged were allowed to the patients with a head size of more than 36 mm for the conventional THR group and all patients in the DMTHR group. Radiographs were taken at immediate postoperative, at six months, and twelve months; and were evaluated for dislocation, loosening, lysis, and migration.

### Statistical analysis

Data recording was done using Microsoft Excel. Descriptive statistics for quantitative data were done using mean ±SD and median and compared with the unpaired T-test. Qualitative data were represented as frequency and percentages; and were compared with the Chi-square test. Data were compared between the two groups – Dual mobility implant and conventional THR implant groups. Range of motion in degrees was analyzed and compared between two groups with the Mann Whitney U test. The Dislocation rates and Modified HHS were analyzed between the two groups by unpaired T-test, Wilcoxon sign Rank test and Mann Whitney U test based on normality testing. The statistics software used was Medcalc.

## Results

### Study population

With a mean age of 74.48 years, 103 patients with fractured neck of femur were operated on with THR and were available for final follow up at one year. Fifty patients (48.54%) were male, and 49 (47.57%) were operated on the right side. Patients were divided into two groups according to the type of implant used. Fifty-two patients (50.48%) underwent dual mobility THR, and 51 (49.52%) underwent conventional THR. Concerning the preoperative demographic data, operative procedure, and postoperative rehabilitation, both the groups were matched (Table 1).

### Clinical outcome

Mean Harris Hip Score (HHS) of the DMTHR group was 76.37 at three months and 87.02 at the end of one year postoperatively, which was significantly better than the conventional THR group 65.65 at three months and 72.96 at one year postoperatively (Table 2). All the patients had a better functional score at one year as compared to three months postoperatively. DMTHR group had better HHS due to better scores in the activity sections (squatting, sitting cross-legged and tying shoelaces) and motion (range of motion). In the conventional THR group, head sizes of 36 mm, 32 mm, and 28 mm were used in 28 (54.9%), 20 (39.2%), and 3 (5.9%) cases, respectively, whereas in the DMTHR group, 28 mm inner head was used in all cases. Eighty percent of DMTHR patients could squat and sit cross-legged versus 55% of the conventional THR patients (with head size more than 36 mm). The mean range of motion for the DMTHR group at 1-year postoperative was 108.65° in flexion, 10° in extension, 28.27° in adduction, 40.77° in abduction, 16.92° in internal rotation, and 40.85° in external rotation; being significantly better than conventional THR group which was 93.53° in flexion, 5° in extension, 23.82° in adduction, 33.33° in abduction, 12.35° in internal rotation, and 37.65° in external rotation. Comparative results are enlisted in Table 2.

**Table 2:** Table showing the comparison in the functional score between the two groups.

Variable	Conventional THR	Dual Mobility THR	P-value
Harris Hip Score at 3 months follow-up at 1-year follow-up	65.6 ± 6.9	72.9 ± 6.7	<0.0001
	76.3 ± 7.7	87.1 ± 6.1	<0.0001
Sitting cross-legged and Squatting			
Number of patients allowed to do	28 (pt with 36 mm head)	52 (all)	<0.0001
Number of patients able to do	17 (61%)	42 (81%)	
Range of Motion at 1-year follow-up			
Flexion	93.5 ± 7.4	108.6 ± 10.7	<0.0001
Extension	5.6 ± 1.6	10.3 ± 2.3	
Abduction	33.3 ± 5.1	40.7 ± 2.8	
Adduction	23.8 ± 4.3	28.3 ± 2.7	
Internal rotation	12.3 ± 5.6	16.92 ± 2.9	
External rotation	37.6 ± 9.6	40.9 ± 2.9	
Postoperative complications			
Dislocation	0	0	<0.0001
Revision THR	0	0	
DVT/PE	0	0	
Deep infection	0	0	
Superficial infection	1	1	
UTI	1	1	

### Radiological outcome

Mean cup inclination on the anteroposterior view was 43.3° in the DMTHR group which was not significantly different from 41.9° of the conventional THR group. The radiological follow-up did not show any signs of loosening, radiolucent lines or heterotrophic ossification in any patient.

### Complications

All patients were allowed to walk with full weight-bearing and climb stairs by three months postoperatively. None of the patients had any complications intraoperatively. None of the patients had postoperative dislocation or required revision arthroplasty. On the first follow-up, two weeks postoperative, one patient from each group was diagnosed with a superficial infection at the incision site and was resolved completely with debridement and antibiotics. One patient from the conventional THR group had a postoperative urinary tract infection, which was resolved with oral antibiotics. All the patients went back to routine daily activities by 1-year postoperatively.

## Discussion

In the present study, the dual mobility group showed significantly better functional outcome of HHS at both the follow-ups than the conventional implant group (*p* = 0.0001). There is some evidence [31–33] in line with this finding. On the other hand, systematic review and metanalysis by Canton et al. [27] and most of the literature on DMTHR [10, 11, 28, 29, 31, 34] have either not been compared or have documented functional outcome equivalent to that with the conventional THR. The score was specifically better in activities sections like sitting cross-legged and squatting and in the motion section. Also, both the groups showed better function at the second follow-up compared with the first of the respective group (*p* = 0.0001), indicating there were no “late dippers” or delayed complications.

The present study has separately compared the postoperative range of motion of the hip joint in these two groups, which, to our best knowledge, has never been done before and has shown DMTHR to be significantly better than conventional THR. This could be due to the design of a dual mobility cup where a smaller inner head provides most of the effective movement. At the extremes of movement on the inner diameter, the femoral neck abuts the outer femoral head and causes this larger head to articulate with the acetabular component. This, therefore, decreases the incidence of impingement and increases the range of movement [31, 35]. This also supports our finding of better scores in squatting and sitting cross-legged activities that need extremes of the range of movements.

A meta-analysis including 3720 patients by Meek et al. [36] has shown postoperative dislocation after primary THR. It is a dreaded complication with a multifactorial etiology and an incidence of 1% in one month, 1.9% in one year, and a constant increase of 1% every 5 years thereafter. The dislocation rate of THR done for the acute fracture neck of the femur in the early postoperative period is 3.9%, which is 2–4 times more than that of the elective THR [36, 37]. Concerning the surgical approach, the posterior approach is associated with a dislocation rate of 3–8% [37, 38]. Meticulous capsular closure and preservation of pyriformis muscle may reduce the incidence to 1% or less [38], but it remains higher than that with the anterior and lateral approach, which is 0.5–0.6% [13]. The head diameter also influences the postoperative dislocation rate in inverse relation. Increasing the head size from 28 mm to 32 mm and 36 mm reduces the dislocation rates from 1.1% to 0.7% and further to 0.5%, respectively [13].

Some patients are at “high risk” for dislocation after THR associated with epidemiological factors such as old age, impaired cognition, non-compliance to rehabilitation protocol, neuromuscular disorders, and patients with a small acetabular cup that does not allow a large femoral head [39]. There is evidence that bipolar hemiarthroplasty may provide better stability in the fracture neck of the femur [40, 41] and is therefore used by many surgeons in such high-risk patients. The dual mobility design of the implant attempts to lower the postoperative instability by a high femoral head-to-neck ratio by allowing articulation between the metal component and the outer polyethylene bearing, known as the “McKee-Farrar” principle [10, 42], which increases the jump distance required for the femoral head to separate from the acetabular component. The increased jump distance decreases the risk of dislocation when the femoral head or neck impinges on the acetabular component [31, 35]. However, except a few [43, 44], most of the literature evidence [2, 45–47] has shown that THR, with Charnley’s principle of low friction arthroplasty, is significantly better than bipolar hemiarthroplasty in terms of long-term functional outcome. Hence, with a combination of these two principles, DMTHR would be an implant to offer the benefits of both [29].

Neri et al. [18], in an original designer retrospective study on Primary DMTHR for 212 cases, showed excellent implant stability and survivorship with no dislocation at a mean follow-up of more than twenty-five years. Zagorov et al. [34] in 2018 compared postoperative dislocation rate with Dual mobility cup with that of conventional THR and Bipolar Hemiarthroplasty for displaced femoral neck fractures and concluded that DMTHR had 0% dislocation rate as compared to 11.1% in conventional THR and 3.1% in bipolar hemiarthroplasty. Similar comparative studies in the literature [10, 11, 27–31] have shown the superiority of DMTHR over conventional THR in terms of postoperative stability and requirement of revision arthroplasty and are enlisted in Table 3. In the present study, no difference was found between postoperative dislocation rate, postoperative stability, implant loosening, and rate of revision surgery between the two groups, as none of the patients had any such complication. Factors contributing to this inconsistency could be lateral approach, use of 36 mm head in majority of conventional THR patients, meticulous capsular closure, exclusion of dysplastic patients and patients with neuromuscular disorders.

**Table 3:** Table showing the comparative studies evaluating the difference in dislocation rates and functional outcome between the DMTHR and conventional THR.

Study	Year	Sample size	Mean follow-up	Mean age in years	Difference in dislocation rate (*P*-value)	Difference in functional outcome (*P*-value)
Tarasevicious et al.	2010	98	1 year	70.8	0.01	>0.01
Tarasevicious et al.	2013	125	1 year	75	0.01	>0.01
Rowan et al.	2017	272	3 years	48.5	0.01	>0.01
Zogorov et al.	2018	116	32 months	73.4	0.05	NA
Present study	2019	103	1 year	76.25	>0.01	0.001

Reluctance in orthopaedic surgeons for dual mobility design was due to previous reports of excessive polyethylene wear (due to dual articulation) [29, 30], aseptic loosening, and intra-prosthetic dissociation with older designs that utilized conventional less dense polyethylene [17, 19], smaller retention collar, and wide rough neck design [18, 48–50]. Newer generations of DM articulations have sought to address these issues by incorporating ultra-high molecular weight polyethylene (UHMWPE), improved capture mechanisms with larger retention collar, optimal neck design with mirror polished surface [18, 48–50], modular coupling options, non-hemispherical shells, and porous coatings [51–53]. This reduces wear particle generation, improves versatility, reduces soft tissue impingement and improves construct stability [51–53]. Dual mobility implants increasingly gain recognition in the literature as an effective option to increase postoperative function, survivorship and reducing instability [10, 11, 40, 41, 43, 44, 50, 54]. However, the fact that there are very few long-term studies available has put a limit to its use.

The strengths of this study are matched groups for comparison and separate assessment of the postoperative range of motion. In contrast, the limitations being the small sample size and short duration of follow-up.

## Conclusion

The present study concludes that dual mobility implants give better results than conventional single-bearing implants for primary THA in elderly patients of fracture neck of femur in terms of better function and greater range of motion. More long-term multicentric studies are still needed on the same line.

## Conflicts of interest

The authors declare that they have no conflicts of interest.

## Funding

This research did not receive any specific funding.

## Ethical approval

Ethical approval was not required.

## Informed consent

This article does not contain any studies involving human subjects.

## Authors contribution

Dr Sanjay Agarwala, Dr Ameya Katariya, Dr Mayank Vijayvargiya, Dr Vivek Shetty, Dr Pravin Manohar Swami have contributed equally to the conception, design, drafting, acquisition, analysis and interpretation of data of the study. All authors read and approved the final manuscript
